# The Trials of the Veterinary Examining Board1Presented at the Fourteenth Annual Meeting of the Iowa State Veterinary Medical Association, Des Moines, Iowa, February 11 and 12, 1902.

**Published:** 1902-05

**Authors:** H. E. Talbot

**Affiliations:** Des Moines, Iowa


### THE TRIALS OF THE VETERINARY EXAMINING BOARD.[1]

By H. E. Talbot, M.D.C.,
DES MOINES, IOWA.

I have been making a few observations lately, and I have noticed that it makes little difference how many roses are thrown in your pathway, if you attempt to pick them up and pin them on your coat you are going to get scratched almost every time. The State Veterinary Examining Board has received its share of roses. We have individually and collectively been congratulated upon our appointment, and we have been complimented for the so-called masterly manner in which we have coped with numerous problems. We have at times come to the conclusion that we had the undivided support of the profession throughout the State, and just as we have resolved to gather together a few of these roses of compliment and bind them into a bouquet of self-congratulation, we have been rudely pricked by hidden thorns of censure or by the disapproval of those who, while condemning our course, have no remedy to offer with which to dress our wounds.

We have been forced to deal with problems which we little expected to encounter in the beginning. We have had no precedent to follow, and have found it necessary to establish precedents as we went along. We have met at times when our treasury was so depleted that we could not definitely set the date of the next meeting, and the overshadowing burden of our thoughts, in the language of Shakespeare, was "When shall we three meet again?"

Presented at the Fourteenth Annual Meeting of the Iowa State Veterinary Medical Association, Des Moines, Iowa, February 11 and 12, 1902.

When we first received our appointment and proudly donned the toga of office we were convinced that we had but one path to follow, and that was the one clearly mapped out for us by the law as passed. Since that time, however, we have discovered how seriously we were mistaken. We have had at least a dozen different interpretations of every section of the law, and the queer part of the whole proceeding is that each interpreter expects us to agree with him. We have been flattered and threatened, cussed and discussed, and assured that we would make a grave mistake if we either granted or refused to grant a certain certificate. In short, we have been made to feel that we were three of the most partial, irresponsible, and generally incompetent men in the State of Iowa.

Our work, however, has not been wholly without recompense. At our meetings we have been delightfully entertained during our moments of leisure by the rich soprano voice of our worthy President, aided by the deep bass voice of our genial Treasurer. On numerous occasions passers-by have been attracted by their masterly renditions of Dr. Heck's favorite, "The Bull-dog on the Bank and the Bull-frog in the Pool," or have stopped to listen to the silvery tones of Dr. Johnston as he led in "My Bonnie Jean." The doctor has always regretted that he didn't study music instead of veterinary medicine, and a number who have heard him sing have regretted it, too. As for myself, I never stop smoking long enough to sing.

We have received a number of very interesting and somewhat humorous letters. Among them was one from an old gentleman, which ran thus: "I am the best horse-doctor in Allamakee Co. Send me a certificate by return mail." Another gentleman, when told that he was not eligible to registration, wrote and asked if $25 would get him a certificate. Of course, you do not expect us to read our answer to him right out loud. Another gentleman admitted that he wasn't eligible, but assured us that if we didn't give him a certificate any way he would expose us in some of our meanness. Now, wasn't that mean? We didn't know what to say to him either. In fact, it is hard to dispose of some of these knotty questions to an advantage and with profit to yourself.

We have been severely condemned in some quarters for acting upon the decision of Judge Holmes, of this district, and issuing certificates to those who were entitled to them at the time the law went into effect. A number have not yet heard of this decision, and in one case proceedings were begun against a man who had been registered and held a certificate under this decision, but the matter was brought to our attention in time to save costs to both parties. As no doubt you are all aware, the decision referred to is to the effect that all non-graduates who were eligible to registration at the time the law went into effect are still eligible upon making the required application, accompanied by affidavits and fee.

All kinds of stories have been afloat regarding this decision—some to the effect that the law was unconstitutional and could not be enforced; and even registered men throughout the State have anxiously written, asking if these stories are true. The worst difficulty which we have encountered, however, has been with the cases of a number who were rejected before and make application again under this ruling. We have watched them, and I think we can safely say that practically none who has been registered under this ruling is incompetent or ineligible.

I have heard somewhere that brevity is the soul of wit, and, as I can readily believe that such a quality would be appreciated at this time, I will draw my paper to a close after reading a short contribution from the pen of the board stenographer. He has been in our employ for so long that he wants to register, but we have told him that treating an average of one case every two years is not sufficient, and that he would have to go to school. The following verses will be appreciated as an illustration of what the veterinarian gets in this world and what he may expect to get in the next :

He's a man who gets up early in the morning,
And the pleasures of good rest he'll never know.
He enjoys a midnight drive out in the country
When the mercury is forty-five below.
He exists because his neighbors want to use him,
He's the slave of all the country, you can bet ;
He's the man you send for quick,
When your hoss is gettin' sick ;
He's that easy-goin' feller called the " vet."

If you're figurin' on gettin' help for nothin',
He's the man you want to call on every time ;
He can work as hard as any man a-livin',
But collectin' doesn't seem to be his line.
He's too busy to remember what you owe him,
And he's mighty glad to take what he can get,
He don't look for any pay
'Til along 'bout judgment day—
And he's seldom disappointed, is the " vet."

When accounts on earth have been marked off the ledger,
And we're all a-takin' chances in the sky,
When we're kinder blockin'up the golden stairway
'Til the angels have to crowd a-gettin' by;
I'll just bet St. Peter 'll come and tell us fellers
That he hasn' any vacant rooms to let,
That we'll have to turn aroun',
Take the elevator down
And go live out in the stable with the "vet."

---

ETIOLOGY OF LAMINITIS. Aruch, Director of the Roumanian Tramways Omnibus Society, has most constantly observed laminitis in horses used for active emergency service, those in good condition and fed strongly on nitrogenous food, those working after several days' rest, and in colts well nourished during breaking. The disease increased or decreased according to the good or poor condition of the animal.

Aruch experimented with the toxic coefficient of the urine in the healthy horse upon rabbits. This coefficient augmented with work, but after rest became normal. This experimental horse was required to perform work producing much fatigue and was then rested for two days. The urine during these two days had a high toxic power and, as Aruch prognosticated, the horse became affected with founder. After feeding hay and grass, or grass alone, and disinfecting the intestinal tract, as the toxic coefficient become less so the symptoms improved, and when the animal was cured the toxic coefficient was normal.

From these phenomena the author concludes that laminitis is a species of auto-intoxication from increased metabolic changes in a system surcharged with nutritious material, as indicated by the increased toxic properties of the urine.

Conclusions: 1. Founder should be considered as the result of a special auto-intoxication.

2. The augmentation of the toxic properties of the urine precede the appearance of the disease.

3. All remedies which stimulate the emunctories, liver, kidneys, skin, etc., and especially a low diet and intestinal disinfection, are indicated in the treatment of founder.—*Annals de Med. Vet.*

